# Age- and sex-patterns of suicide trends in Europe: 1990–2022 comparative analysis of official WHO mortality data

**DOI:** 10.1192/j.eurpsy.2025.10137

**Published:** 2025-12-02

**Authors:** Paola Bertuccico, Andrea Amerio, Giansanto Mosconi, Enrico Grande, Carlo La Vecchia, Alessandra Costanza, Giacomo Pietro Vigezzi, Riccardo Vecchio, Andrea Aguglia, Isabella Berardelli, Gianluca Serafini, Mario Amore, Maurizio Pompili, Anna Odone

**Affiliations:** 1 Department of Public Health, Experimental and Forensic Medicine, University of Pavia, Pavia, Italy; 2 Department of Neuroscience, Rehabilitation, Ophthalmology, Genetics, Maternal and Child Health (DINOGMI), Section of Psychiatry, University of Genoa, Genoa, Italy; 3 IRCCS Ospedale Policlinico San Martino, Genoa, Italy; 4 Integrated System for Health, Social Assistance and Welfare, National Institute of Statistics, Rome, Italy; 5 Department of Clinical Sciences and Community Health, University of Milan, Milan, Italy; 6 Department of Psychiatry, Faculty of Medicine, Geneva University (UNIGE), Geneva, Switzerland; 7 Department of Psychiatry, Faculty of Biomedical Sciences, University of Italian Switzerland, Lugano, Switzerland; 8 Department of Neurosciences, Mental Health and Sensory Organs, Faculty of Medicine and Psychology, Suicide Prevention Centre, Sant’Andrea Hospital, Sapienza University of Rome, Rome, Italy; 9 Medical Direction, Fondazione IRCCS Policlinico San Matteo, Pavia, Italy

**Keywords:** adults, elderly, mortality, suicide, young

## Abstract

**Background:**

Understanding how suicide rates vary across age, sex, and geography is essential to designing effective prevention strategies. We examined long-term trends in suicide mortality across European countries over three decades, with a focus on age-specific trajectories.

**Methods:**

Using the WHO mortality database, we computed annual sex- and age-specific suicide rates (10–14 to 85+ age groups) from 1990 to 2022, for the most populous European countries, and aggregated rates for the EU-27 and four geographical areas (North, West, South, and Centre-East Europe). We also calculated percentage differences across four time periods (1990–1994, 2000–2004, 2010–2014, and 2020–2022), according to data availability.

**Results:**

Suicide rates increased with age, peaking in older individuals (85+) in most countries (e.g., 82.0/100,000 in France in 2020–2022, 77.1/100,000 in Germany among males, in 2020), except in the UK and Northern Europe, where rates peaked at middle age (∼22/100,000 at 45–49, in 2020). EU-27 suicide rates in 2020 ranged from 5.5/100,000 (age 15–19) to 58.2/100,000 (85+) among males, and from 2.6 (15–19) to 8.6/100,000 (85+) among females. Male suicide rates were 3 to 8 times higher than female rates across all ages. While overall rates declined since 1990 in most countries, youth suicide increased after 2010 in Western (e.g., +12%, girls 15–19), Southern (+24.5%, girls 15–19), and Northern (+44%, girls 15–19 and 20–24) Europe. Rates among young and middle-aged adults recently rose in Spain, the UK, and Northern Europe, while they declined in Eastern Europe after the 1990s.

**Conclusions:**

Despite overall declines, our findings highlight marked heterogeneity in sex- and age-specific trends in suicide mortality across Europe. These patterns call for age-tailored prevention strategies that address evolving psychosocial stressors and structural determinants across the lifespan.

## Introduction

Suicide is a major public health issue worldwide and its prevention — a key priority of the United Nations Sustainable Development Goals, the World Health Organization’s (WHO) General Programme of Work, and the Comprehensive Mental Health Action Plan 2013–2030 — is receiving increasing attention even in countries where suicide rates are less alarming [1].

Contrary to popular belief, suicide is neither a normal response to the levels of stress experienced by the majority of people nor a linear consequence of major psychiatric disorders [2]. Suicide is among the most serious warning signs in both everyday life and psychiatric practice, and one of the most frequent reasons for referral to mental health services.

According to the Global Burden of Disease (GBD) estimates, 746,000 deaths (95% uncertainty intervals, UI: 692,000–800,000) from suicide were estimated in 2021 worldwide, including 519,000 deaths (485,000–556,000) among males and 227,000 (200,000–255,000) among females [3]. However, only a few comparative studies have systematically examined long-term age- and sex-specific suicide mortality trends across European regions. While recent global studies reported overall declines in suicide mortality [4] and some focused analyses examined trends among specific age groups as young adults [5], there is still a lack of long-term, age- and sex-disaggregated analyses across the lifespan in Europe. The risks of suicide vary significantly across the lifespan, with age and gender playing a particularly important role. Concerning the age group 10–24 years, notable geographical disparities worldwide in suicide rates and trends emerged over the past 30 years, with males showing rates 2 to 5 times higher than females [6]. While most European countries reported favorable trends, with the exception of the UK, rising suicide rates were observed in the USA as well as in most Central and Latin America and Australasia. Focusing on the age group 75 years or older, while declines in suicide rates were observed in many countries worldwide over the past three decades, recent trends indicate a slowing of those declines (Germany, Italy, the Netherlands, Spain, and the UK), with some regions experiencing increases (USA, Brazil, and Argentina) [7]. The oldest age group (85+) consistently exhibited the highest suicide rates, particularly among males, highlighting their vulnerability. Additionally, although a sex ratio M/F of 3–7:1 was observed, a narrowing of the sex gap in mortality levels was noted in some regions of Western and Southern Europe, as well as in Argentina.

According to the recent literature, the age groups 10–24 and 75 years or older are not the ones to be paid most attention to in terms of suicide risk [8]. Aware of this, based on the official WHO mortality database, the current study aims to analyze age- and sex-specific patterns in suicide mortality across the lifespan (from 10–14 to 85+ age groups) among European countries, covering the period from 1990 to 2020 or the most recent available year.

## Methods

We extracted data on official suicide death counts and population estimates by five-year age groups (i.e., from 10–14 to 80–84, plus 85+), disaggregated by sex, country, and calendar year, from 1990 to the most recent available, using the publicly accessible World Health Organization (WHO) mortality database [9]. When population data were missing in the WHO database (i.e., for some calendar years), we extracted them from the United Nations database [10], which provides age- and sex-specific population estimates in the same five-year age group structure, ensuring consistency with the WHO classification. When mortality data were missing for specific years, no interpolation was performed, since our analyses relied exclusively on official statistics.

Suicide deaths were identified using the codes from three successive revisions of the International Classification of Diseases (ICD) applied during the study period: ICD-8 and ICD-9 codes E950–959, and ICD-10 codes X60-X84, Y87.0.

Out of 140 countries included in the WHO mortality database, we focused on the European countries. We selected countries according to the following inclusion criteria: i) population size greater than 35 million, to maximize representativeness and reduce random variability in age-specific data; and ii) completeness of mortality data of over 95%, to minimize bias from underreporting and ensure comparability across countries. Thus, we included: France, Germany, Italy, Poland, the Russian Federation, Spain, Ukraine, and the United Kingdom (UK). Additionally, we aggregated data for the European Union (EU-27) as a whole (27 Member States, excluding Cyprus for which data were available only for a limited number of years), and for four geographic areas: 1) Northern Europe, including Denmark, Finland, Iceland, Ireland, Norway, Sweden, and the UK; 2) Western Europe, including Austria, Belgium, France, Germany, Luxembourg, the Netherlands, and Switzerland; 3) Southern Europe, including Greece, Italy, Malta, Portugal, and Spain; 4) Central-Eastern Europe, including Belarus, Bulgaria, Croatia, Czech Republic, Estonia, Hungary, Latvia, Lithuania, North Macedonia, Poland, Republic of Moldova, Romania, Serbia, Slovakia, Slovenia, and Ukraine.

For each country or geographic area, sex, and calendar year, we computed age-specific mortality rates per 100,000 person-years, from the youngest age group (10–14 years) to the oldest ones (85+ years). We then provided country-, age- and sex-specific numbers of deaths and corresponding rates for four time periods over three decades: 1990–1994, 2000–2004, 2010–2014, and 2020–2022. These periods were chosen to maximize data availability across countries, reduce the influence of single-year fluctuations (especially in small age groups), and capture long-term decadal changes while highlighting the most recent years. We computed and graphically displayed the corresponding percentage differences: 1990 (1990–1994) versus 2000 (2000–2004), 2000 (2000–2004) versus 2010 (2010–2014), and 2010 (2010–2014) versus 2020 (2020–2022), separately by country and sex.

For the analyses, we used the software SAS version 9.4 and the software R version 4.1.1.

## Results

### Suicide mortality rates by age

Table 1 gives age-specific suicide mortality rates in 2020–2022 (or according to data availability) for each country, and in 2020 for geographical areas, separately by sex. The same data are also presented in Supplementary Figures S1 and S2 as pyramid-style plots, providing a clearer visualization of sex- and age-related patterns.

**Table 1.** Age-specific suicide mortality rates since 2020*, by country or geographical area, and sex

		Age 10–14	Age 15–19	Age 20–24	Age 25–29	Age 30–34	Age 35–39	Age 40–44	Age 45–49	Age 50–54	Age 55–59	Age 60–64	Age 65–69	Age 70–74	Age 75–79	Age 80–84	Age 85+
France	Males	1.0	4.8	10.9	14.1	18.2	22.2	23.7	31.0	33.2	30.6	26.0	26.3	24.5	35.5	52.9	82.0
	Females	0.3	2.3	3.5	4.0	4.6	4.5	6.3	8.8	11.7	11.8	9.8	9.7	8.5	11.4	12.1	10.0
Germany	Males	0.7	5.2	10.8	11.3	11.9	14.6	15.0	15.3	19.9	20.8	22.1	23.3	23.7	30.9	48.4	77.1
	Females	0.6	2.7	3.1	3.1	3.4	4.2	4.5	5.1	7.1	8.4	6.4	5.7	6.5	10.6	10.2	13.9
Italy	Males	0.5	4.1	6.5	7.5	8.4	9.1	9.9	11.7	11.4	13.6	13.6	13.7	14.1	17.7	23.0	33.6
	Females	0.5	2.6	1.8	2.3	2.6	2.5	2.7	3.5	3.0	3.9	3.1	3.3	3.6	3.6	3.4	4.0
Poland	Males	0.8	10.4	22.6	23.6	25.5	24.5	25.9	25.4	28.5	34.0	31.2	24.9	23.7	27.1	26.7	40.4
	Females	1.2	5.2	2.3	2.9	3.1	3.0	2.5	3.6	4.2	5.0	4.4	5.9	4.1	3.3	4.3	4.8
Russian Federation	Males	1.3	10.7	19.6	23.0	26.8	28.1	29.1	25.4	25.8	27.3	23.6	28.4	31.1	43.6	44.5	52.6
	Females	1.0	4.0	2.9	3.1	3.9	3.6	3.9	4.0	3.9	3.2	3.1	3.5	4.9	8.3	9.1	16.3
Spain	Males	1.1	2.2	7.7	8.8	10.4	11.1	14.6	16.0	17.3	17.9	18.0	13.3	15.6	24.5	31.9	42.7
	Females	0.7	2.1	2.6	1.9	3.1	3.6	3.9	6.0	7.3	5.9	5.5	5.7	4.6	6.3	7.6	5.6
Ukraine	Males	1.2	11.0	20.8	24.1	29.5	31.4	32.3	33.2	30.5	38.4	38.0	35.1	35.0	57.3	55.5	65.4
	Females	0.8	4.0	2.9	4.5	5.0	3.8	4.8	4.8	4.9	4.8	4.8	6.4	5.3	10.1	11.5	15.5
UK	Males	0.5	6.6	12.6	14.8	17.5	20.0	17.5	22.1	19.4	16.4	14.2	11.0	10.0	8.8	8.8	15.1
	Females	0.4	2.7	4.3	5.3	4.6	5.7	5.2	6.5	6.5	5.3	4.8	3.0	2.6	2.3	3.7	3.2
EU (27)	Males	0.7	5.5	11.4	13.5	14.6	16.6	17.8	19.6	22.1	22.8	22.6	22.1	21.7	28.5	40.8	58.2
	Females	0.6	2.6	3.2	3.4	3.5	4.1	4.6	5.8	7.2	7.5	6.8	6.1	6.3	7.6	8.6	8.6
North Europe	Males	0.6	7.1	13.5	15.6	17.4	19.8	18.1	21.7	19.8	17.3	15.5	12.3	11.7	10.1	12.1	18.8
	Females	0.5	3.2	5.0	5.7	4.8	6.1	5.6	6.6	7.1	5.6	5.6	3.9	3.3	2.9	4.0	3.5
West Europe	Males	0.9	5.4	11.0	12.5	14.7	17.9	18.7	22.1	24.9	24.7	23.7	23.9	23.5	32.1	48.0	74.5
	Females	0.7	2.9	3.8	3.8	4.2	4.6	5.6	7.4	9.3	9.6	8.3	7.7	7.8	10.5	11.0	11.5
South Europe	Males	0.4	2.7	6.2	7.6	8.7	9.4	11.4	12.6	14.0	14.3	13.8	13.2	14.2	19.8	25.6	36.2
	Females	0.3	1.5	1.4	1.9	2.1	2.8	3.3	4.0	4.9	5.1	4.1	3.6	4.0	4.3	5.0	4.8
Centre-East Europe	Males	0.5	8.2	16.0	19.6	18.8	20.8	22.7	23.4	27.6	30.5	32.7	31.0	30.2	42.7	54.6	65.0
	Females	0.8	3.0	2.4	2.8	2.9	3.6	3.7	5.0	5.0	6.1	6.5	5.7	6.7	7.6	9.4	10.3

*Note:* *Calendar years available: 2019 for the Russian Federation and Ukraine; 2020 for Germany, the UK, the EU (27), North Europe, West Europe, South Europe, and Centre-East Europe; 2020–2021 for Italy; 2020–2022 for France, Poland, and Spain.

Among males, suicide mortality rates increased with age in all the countries considered, although the rise was not linear and tended to show a plateau during mid-adulthood, mainly in males. Excluding the youngest age group (10–14 years), in France and Germany, rates rose from about 5/100,000 among adolescents (age group 15–19) to over 75/100,000 in the oldest age group (85+). Italy showed a similar age-related increase but with lower absolute levels, peaking at around 34/100,000. In Poland, the Russian Federation, and Ukraine, rates also increased steadily across age groups, ranging from around 10–11/100,000 at ages 15–19 to 40.4, 52.6, and 65.4/100,000, respectively, in the oldest age group (85+). In Spain, suicide rates were lower in adolescence (about 2/100,000), reaching 42.7/100,000 in later life (85+). Differently, the UK showed higher suicide rates in the middle age groups (reaching 22.1/100,000 at ages 45–49), followed by lower rates and a modest increase again among the oldest. Regarding geographical areas, in the EU-27, suicide mortality rates increased from 5.5 (15–19) to 58.2/100,000 in the oldest age group. In Northern Europe, rates were high at middle ages (21.7/100,000 at 45–49 years), followed by a decrease and a smaller rise in old age (18.8/100,000 at 85+). In Western Europe, rates increased from 5.4 (15–19) to 74.5/100,000 (85+), in Southern Europe from 2.7 to 36.2/100,000, and in Central-Eastern Europe from 8.2 to 65.0/100,000, over the same age range.

Among females, the rates were lower compared to those of males, but they showed a similar age-related increase. Overall, the variation in suicide mortality across age groups appeared less pronounced among females than among males. Excluding the youngest age group (10–14 years), in the 15–19 age group, female suicide mortality rates were approximately half of those observed in males. The male-to-female gap tended to widen with age in most countries, reaching a ratio of 8:1 in the oldest age group in France, Italy, and Poland. In contrast, in Central-Eastern Europe and in the UK, the gap was already high among the younger and remained relatively stable among adults. Looking at the geographical areas, in the EU-27, female suicide mortality rates increased from 2.6/100,000 at ages 15–19 to 8.6/100,000 at 85+. In Northern Europe, rates were highest during adulthood, peaking at 7.1/100,000 in the 50–54 age group, and then slightly declining with stable levels of about 3.5/100,000 in the oldest age group. In Western Europe, rates increased from 2.9/100,000 at 15–19 years to 11.5/100,000 at 85+, while in Southern Europe, rates increased from 1.5/100,000 at 15–19 years to approximately 5/100,000 from age 50 onwards, remaining relatively stable through the older age groups. In Central-Eastern Europe, rates rose from 3/100,000 at ages 15–19 to about 10/100,000 at 85 + .

### Suicide mortality trends across five-year age groups over time

**Figure 1.**
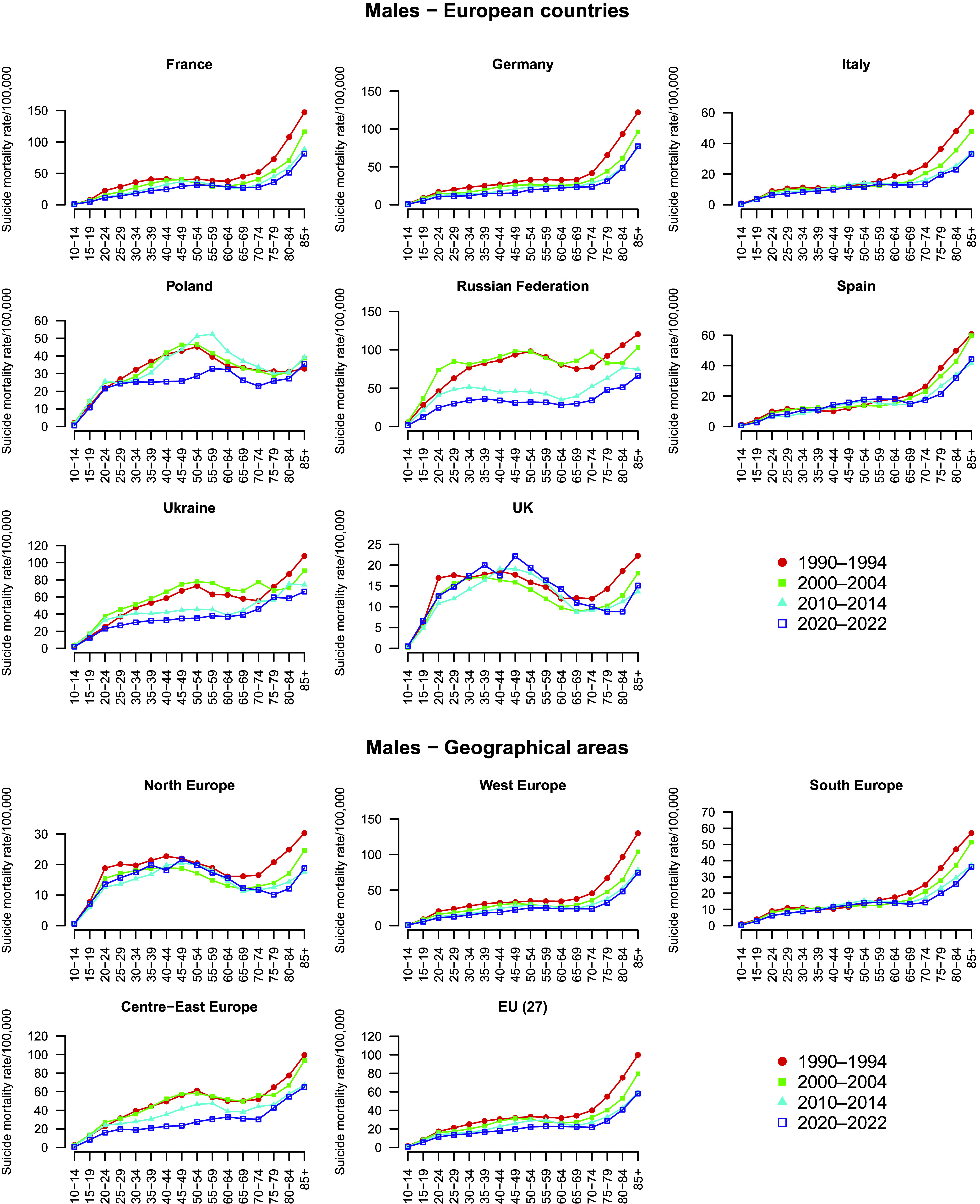
Trends in age-specific suicide mortality rates (per 100,000) among males in four periods (1990–1994, 2000–2004, 2010–2014, and 2020–2022), by country and geographical area. *Note:* For the Russian Federation and Ukraine, 2015–2019 period were used in place of 2020–2022, due to limited availability.

**Figure 2.**
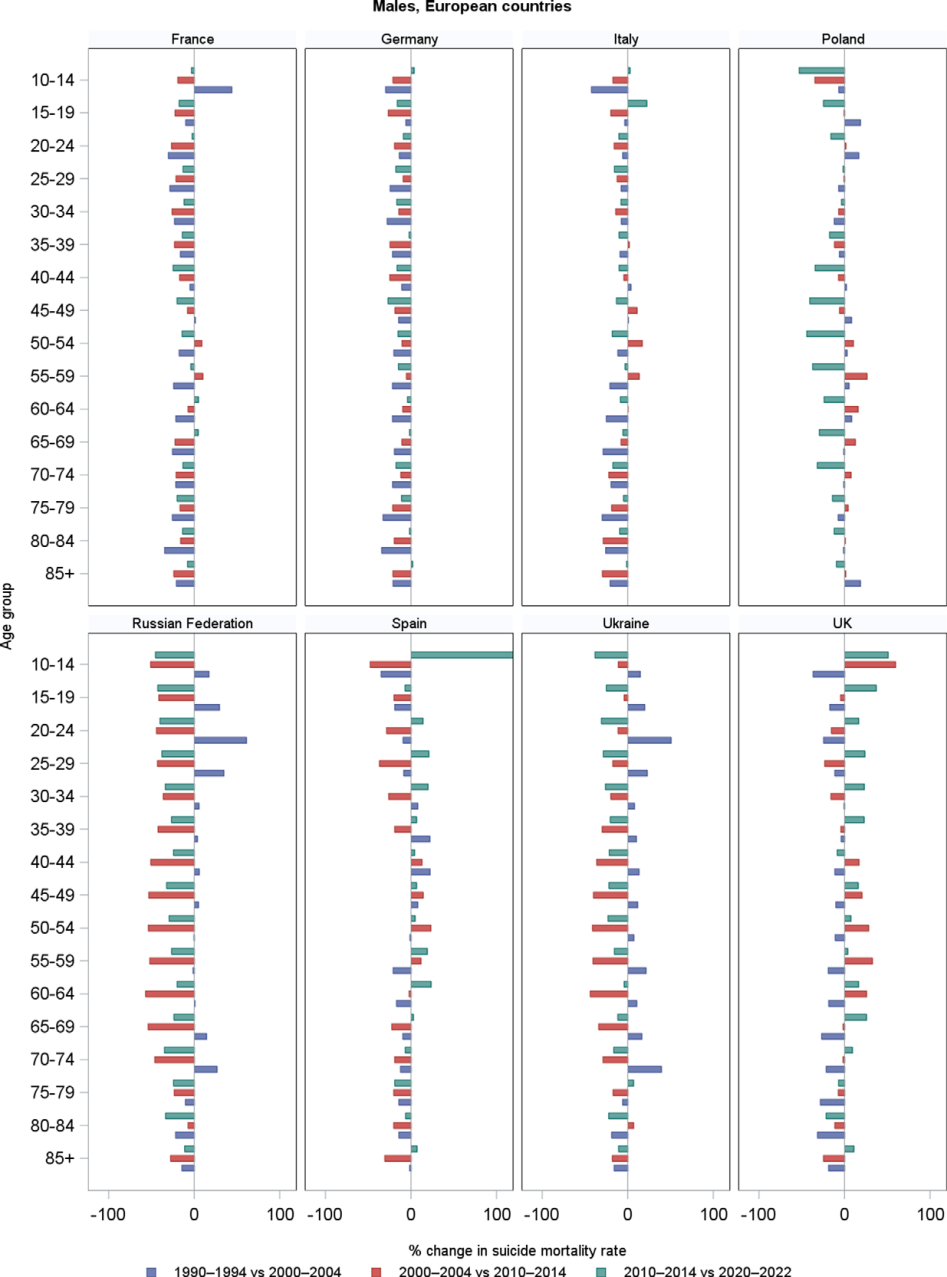
Percentage changes in age-specific suicide mortality rates among males across three consecutive periods: 1990 (1990–1994) versus 2000 (2000–2004), 2000 (2000–2004) versus 2010 (2010–2014), and 2010 (2010–2014) versus 2020 (2020–2022), by country. *Note:* For the Russian Federation and Ukraine, 2015–2019 period were used in place of 2020–2022, due to limited availability.

**Figure 3.**
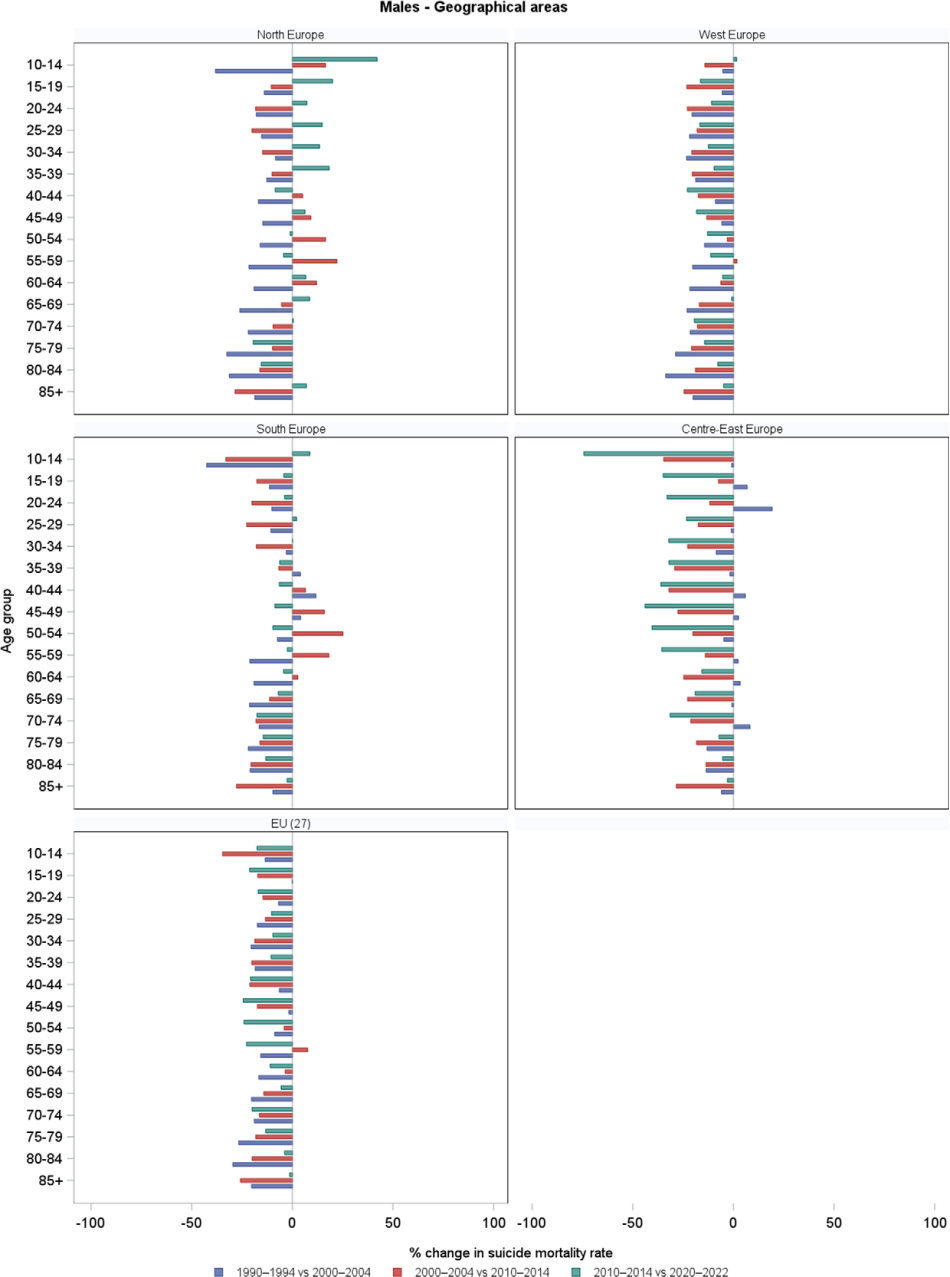
Percentage changes in age-specific suicide mortality rates among males across three consecutive periods: 1990 (1990–1994) versus 2000 (2000–2004), 2000 (2000–2004) versus 2010 (2010–2014), and 2010 (2010–2014) versus 2020 (2020–2022), by geographical area.

Figure 1 displays suicide mortality trends by five-year age groups among males in four different periods: 1990–1994, 2000–2004, 2010–2014, and 2020–2022, separately by country and geographical area. Figures 2 and 3 gives the corresponding percentage changes by country (Figure 2) and geographical areas (Figure 3). Supplementary Table S1 reports all the numbers shown in the above-mentioned figures.

Among males, suicide mortality rates declined over time in most age groups across all countries and areas considered, although with some exceptions, particularly during the first two decades (i.e., 1990–2000 and 2000–2010). The greatest reductions were observed in younger and middle-aged males between 1990 and 2010. In France and Germany, suicide mortality declined consistently across all age groups and decades, especially between 1990 and 2010. However, some decelerations or modest increases were observed in the last decade at selected ages: in France, around +5% at 60–64 and 65–69; in Germany +1.8% at 85+. In Italy, overall suicide mortality declined, especially during the 1990s. However, some increases were observed among adults in males in 2000–2010 (+17% at 50–54), before declining again, and among adolescents in 2010–2020 (+22% at 15–19). The substantial reductions among the elderly observed until 2010 (−30% at 85+) became smaller thereafter (−1.4% in 2010–2020 at 85+). In Poland, suicide rates increased during the 1990s, particularly among males (e.g., +16.5% at 20–24), then declined progressively. A peak was observed at 55–59 around 2000 (+26% in 2000–2010), followed by sharp reductions in the most recent decade across all adult age groups. In the Russian Federation and Ukraine, suicide mortality increased across age groups during the 1990s. Thereafter, rates declined substantially and steadily over the course of two decades. In Russia, the reductions between 2010 and 2020 ranged from −45% to −11% (e.g., −32% at 45–49 years), as well as in Ukraine, although with slightly smaller declines (−25% at 15–19, −9.5% at 85+). In Spain, mortality declined among younger and middle-aged males up to 2010, but increased in several age groups in the most recent decade (e.g., +21% at 25–29, +7% at 85+). In the UK, suicide rates declined up to 2000, but then rose in most age groups, particularly among adolescents (+37% at 15–19), young adults (about +23% at 30–34 and 35–39), middle-aged males (+16% at 45–49), and the elderly (+10% at 85+). In the EU-27 and Western Europe, suicide mortality among males declined steadily across all age groups and decades. In Northern Europe, early declines were followed by increases, notably +18% at 35–39 in the last decade. A general reduction in rates was observed also in Southern Europe, although some increases emerged among adults (40–64 years) between 2000 and 2010. In Central-Eastern Europe, rates increased across some age groups in the 1990s, followed by marked and consistent declines thereafter.

**Figure 4.**
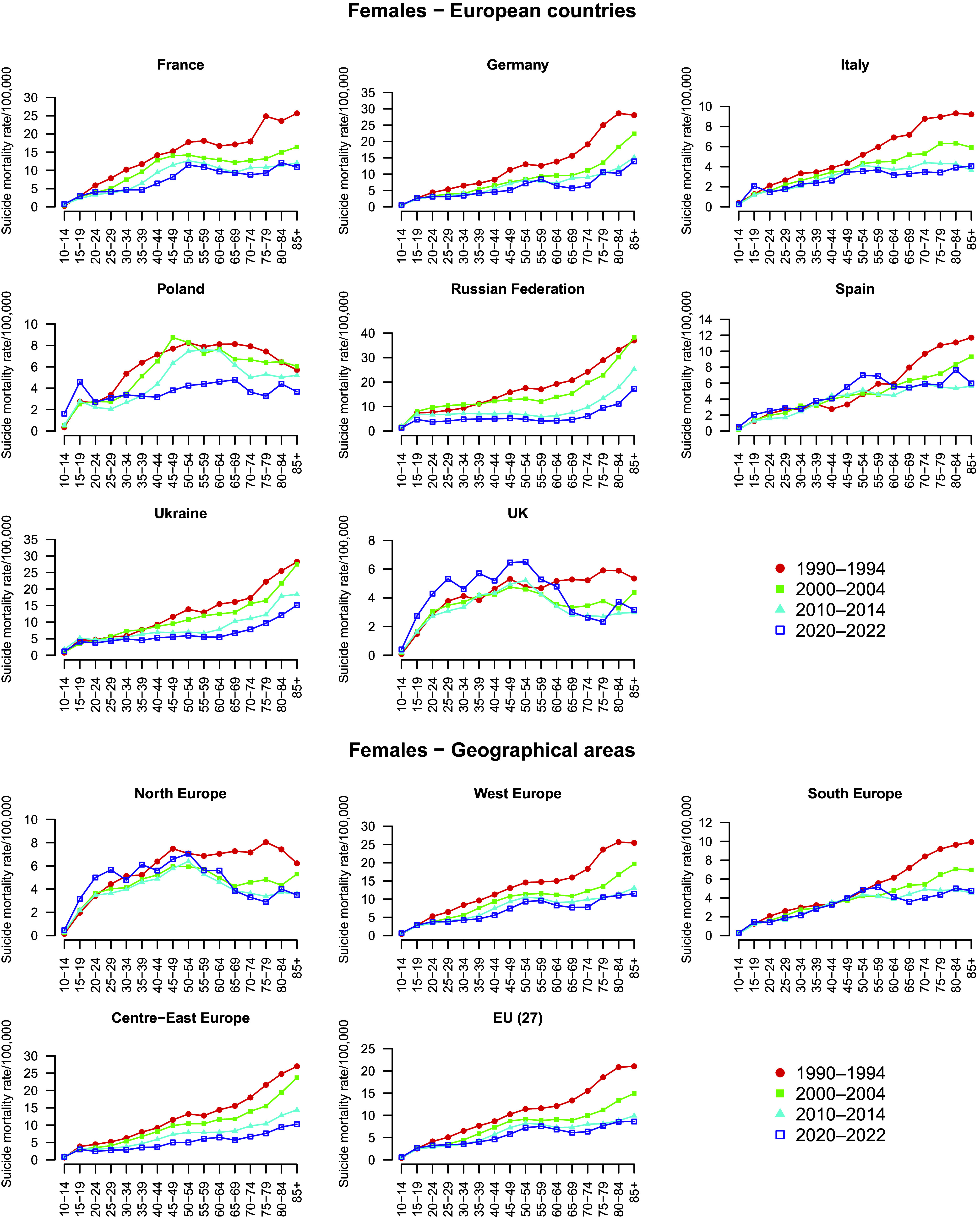
Trends in age-specific suicide mortality rates (per 100,000) among females in four periods (1990–1994, 2000–2004, 2010–2014, and 2020–2022), by country and geographical area. *Note:* For the Russian Federation and Ukraine, 2015–2019 period were used in place of 2020–2022, due to limited availability.

**Figure 5.**
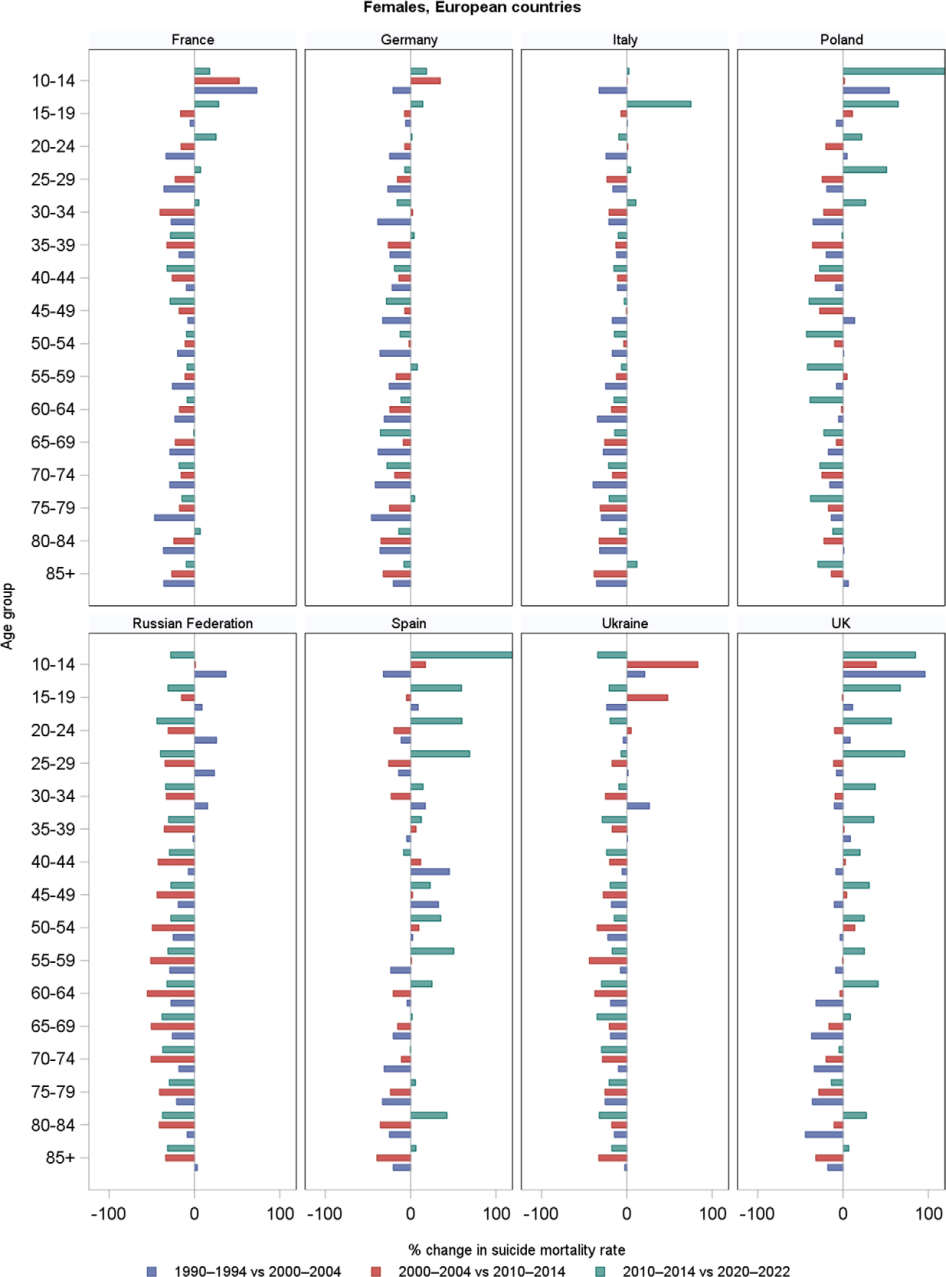
Percentage changes in age-specific suicide mortality rates among females across three consecutive periods: 1990 (1990–1994) versus 2000 (2000–2004), 2000 (2000–2004) versus 2010 (2010–2014), and 2010 (2010–2014) versus 2020 (2020–2022), by country. *Note:* For the Russian Federation and Ukraine, 2015–2019 period were used in place of 2020–2022, due to limited availability.

**Figure 6.**
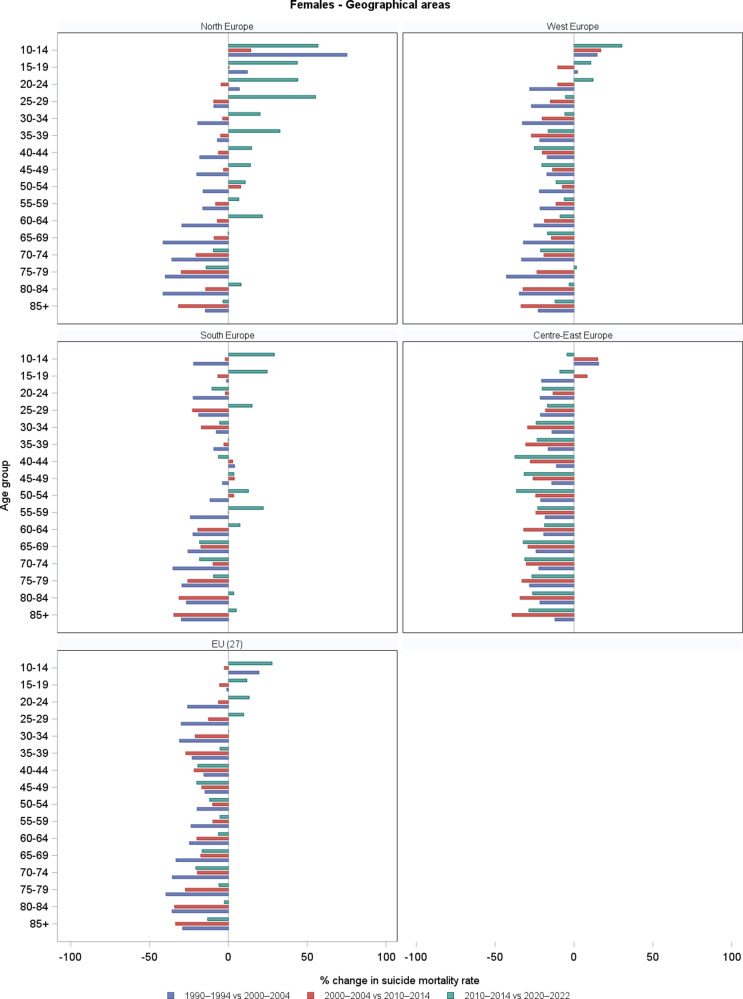
Percentage changes in age-specific suicide mortality rates among females across three consecutive periods: 1990 (1990–1994) versus 2000 (2000–2004), 2000 (2000–2004) versus 2010 (2010–2014), and 2010 (2010–2014) versus 2020 (2020–2022), by geographical area.

Among females, trends in age-specific suicide mortality are given in Figure 4 and corresponding changes in Figures 5 and 6, by country (Figure 5) and geographical area (Figure 6). Supplementary Table S2 gives the corresponding numbers. Suicide mortality rates were generally lower than in males, and trends over time were often more stable or showed less pronounced fluctuations. However, several differences emerged across countries and age groups. In France and Germany, suicide rates decreased over the three decades in most age groups, similarly to males, but some increases were observed in the most recent decade, especially in younger age groups (at 15–19: +28% in France; +14% in Germany), but also in other age groups. In Italy, overall declines were observed across most age groups, but recent increases between 2010 and 2020 emerged among adolescents (+75% at ages 15–19), young adults (+10% at ages 30–34), and females aged 85+ (+12%). In Poland, suicide mortality among females aged under 45 rose in the last decade, with marked increases at 15–19 (+64.5%), and 25–29 (+51%), while declines persisted in the other age groups, especially at older ages. In the Russian Federation and Ukraine, rates increased in the 1990s across age groups but showed steady and substantial declines in the two subsequent decades, mirroring the pattern observed in males. In Spain and the UK, female suicide mortality rose across several age groups in the last decade. Notably, Spain showed recent increases of around +60% at age 15–19 and 20–24, +50% at 55–59 and + 42% at 80–84; the UK +67% at 15–19 and + 72% at 25.29. Considering the geographical areas, EU-27 suicide mortality among females declined in most age groups between 1990 and 2010, but increased slightly or plateaued in the last decade, especially in younger groups (e.g., +13% at 20–24). In Northern Europe, rates increased across several female age groups in the last decade, especially at younger ages (e.g., about +44% at 15–19, +55% at 25–29), and modest rises were observed even among the elderly. Other areas showed continued declines: in Western and Central-Eastern Europe, downward trends persisted across all age groups, apart from some increases in the youngest age groups (e.g. +30% at 10–14 in 2010–2020). Southern Europe showed some recent increases in selected groups, notably +22% in the 55–59 age group.

## Discussion

Our findings on age- and sex-specific suicide mortality trajectories in Europe from 1990 to 2022 reveal a complex and heterogeneous landscape, characterized by an overall decline alongside concerning increases in specific age groups and countries. Suicide rates increased with age, peaking in the oldest age groups in most European countries, except in the UK and Northern Europe, where the highest rates were observed in adults. Male suicide rates were more variable and consistently 3 to 8 times higher than female rates across all age groups. Favorable trends over the period 1990–2010 have been followed by a slowdown or a reversal thereafter among older adults in several Western and Southern European countries. In parallel, increases were observed among younger populations of both sexes in most European countries since the 2010s. Patterns among working-age adults were more variable.

Interpreting suicide mortality patterns requires a broad perspective, drawing on multidisciplinary concepts and a wide range of contributing factors. Suicide and suicidal behavior often represent a maladaptive response to overwhelming mental pain, an intricate interplay of psychological, social, and existential factors, shaped by life-course experiences [2, 11]. Understanding this multifactorial nature is essential to interpreting temporal trends and designing effective prevention strategies across the lifespan.

Our findings align with and expand upon previous analyses [3, 6, 7, 12], while offering more granular insight into European dynamics. Most favorable trends observed likely reflect improved social support and treatment of depressive disorders over the recent calendar periods [13].

A particularly salient feature emerging from our study is the aging of the suicide burden. Suicide consistently increased with advancing age in most countries, reflecting long-standing demographic patterns. According to the GBD 2021 study, the mean age at death from suicide increased from 43.0 years (95% UI 38.0–45.8) in 1990 to 47.0 years (43.5–50.6) in 2021 for males, and from 41.9 years (30.9–46.7) to 46.9 years (41.2–52.8) for females [3]. This age-related gradient likely results from cumulative vulnerability over the life course, compounded by chronic illness, social isolation, and limited access to age-appropriate mental health care in later life, as well as persistent stigma and lack of prevention in mental health.

Concerning recent suicide increases among adolescents and young adults, especially females, in several European countries [6, 14], growing evidence highlights the need for urgent interventions [15]. In England, hospital admissions for mental health conditions among adolescents rose by 65% between 2012 and 2022, with a + 113% yearly increase among girls aged 11–15 [16]. These trends reflect rising levels of unmet psychological needs, likely driven by evolving risk factors such as digital media use, cyberbullying, scholastic or academic stress, eating disorders, and climate anxiety, as well as broader social vulnerabilities [17, 18]. These possible explanations remain speculative in the context of our ecological data, but they are consistent with recent studies reporting associations between such factors and youth mental health outcomes. Social media poses both risks and opportunities: while linked to 24% of youth suicide deaths in the UK (aged 10–19) [17], especially among girls, it also enables prevention through awareness campaigns and online support [19, 20]. Thus, interventions should promote digital education and online safety, rather than restrictions alone. The COVID-19 pandemic likely amplified vulnerabilities, highlighting the need for better data and targeted strategies [21]. However, this interpretation should be viewed with caution, given the limitations of mortality data availability for the 2020–2022 post-pandemic period, and the inability to capture causal mechanisms in our analysis.

Patterns in suicide trends among adults showed some rises, with heterogeneous trajectories. The global 2008 economic crisis most likely significantly impacted working-age populations, as seen in Italy, Spain, and the UK during the second decade [22]. Extensive evidence has linked suicide rates and unemployment and income loss. In Central-Eastern Europe rise during the 1990s–2000s was likely driven by socioeconomic instability of the post-Soviet transition, and widespread alcohol misuse [23, 24]. Comparative evidence from Central European countries confirmed persistently high rates and male predominance over 1990–2019, despite overall declines [25]. Our findings are consistent with these results and extend them to a broader European context, thus facilitating a more comprehensive interpretation of trajectories across the other European areas. Recent increases in Spain, the UK, and Northern Europe may reflect broader stressors linked to the COVID-19 pandemic, such as job insecurity, disrupted routines, reduced support networks, and limited healthcare access [26]. Supporting this, a nationwide Danish study reported increased antidepressant use and a rise in suicide risk during the second lockdown period [27]. Additional pressures from caregiving and work–family conflict further affected midlife populations [28]. Despite limited policy attention, suicide in midlife remains a key concern and requires age-specific, targeted prevention.

Stable or slowing suicide among older adults likely mirrors multiple age-related vulnerabilities [7, 29]. While depression is a key risk factor [30], suicide in later life is also linked to chronic pain, multimorbidity, cognitive decline, loneliness, and barriers to healthcare access. The 2008 economic downturn and the COVID-19 pandemic may have contributed to unfavorable trends. Older adults might experience a cumulative erosion of financial security rather than immediate labour shocks, leading to a plateau in previously declining rates [26]. The pandemic further exacerbated factors such as social isolation, loss of loved ones, and disrupted care [31, 32]. These patterns underscore the need for proper mental health screening in primary care, improving access to care, and strengthened social support.

The findings of this study have significant implications for public mental health strategies in Europe. They highlight the need for tailoring suicide prevention efforts to address demographic and contextual vulnerabilities. While several observed declines are encouraging, recent increases among adolescents and young adults in some countries demand urgent investment in youth mental health, encompassing early intervention, eHealth literacy, and support infrastructures within schools and communities. For working-age populations, priorities include reducing stigma, improving access to care, and promoting outreach in occupational and community settings. Given Europe’s aging population, primary and secondary prevention strategies should also overcome age-related barriers and ensure access to integrated, community-based services that foster connection and autonomy [33]. Enabling supportive and inclusive environments across the whole lifespan (i.e., at school, at university, at the workplace, and after retirement) might promote successful interventions that accompany individuals across crucial and, potentially, challenging life transitions.

Regional disparities suggest the role of structural and cultural determinants, such as welfare systems, healthcare accessibility, and stigma [13]. Differences in suicide trends may partially reflect variations in mental health services provision and social protection systems [34]. Other explanations for the observed cross-national and temporal variations may therefore include differences in mental health care infrastructure, socioeconomic conditions, and policy interventions. However, given the ecological nature of our study, our findings should be interpreted as descriptive associations rather than causal evidence. Ensuring equitable access to care, especially during crises, must remain a core priority. Ongoing surveillance based on disaggregated, high-quality data is essential to monitor evolving patterns and guide responsive, equitable, and effective public health interventions.

This study has several strengths. First, we used official cause-of-death data from the WHO mortality database, ensuring high comparability across countries and consistency over time. Second, the analysis encompasses more than 30 years of data, enabling us to explore long-term patterns across multiple generations and periods, including the 2008 economic crisis and the short-term effects of the COVID-19 pandemic. Interestingly, the granularity of our analysis, by 5-year age groups and sex, allowed us to detect even modest diverging trends and age-specific reversals that would have remained hidden in more aggregated data.

However, several limitations should be taken into account. As in all cross-national suicide research, differences in death certification, underreporting, misclassification, and cultural stigma may affect the comparability of suicide mortality data. Misclassification, especially as undetermined or accidental deaths, may vary by country, age, and sex, potentially leading to underestimation of suicide rates in certain settings. Although we included only countries with high completeness and quality of death certification data, residual inconsistencies cannot be entirely ruled out. Some countries had limited or less recent data availability, particularly in the early 1990s, or post-2020, which may have affected temporal comparability, especially given that the most recent period (2020–2022) covers fewer years and in some countries only up to 2019, 2020, or 2021. To mitigate these issues, we used exclusively official WHO and UN statistics, applied strict inclusion criteria (>95% completeness), and focused on broad temporal and regional patterns rather than precise annual estimates. Finally, as this is a descriptive ecological study, no statistical inference or causal modeling was performed, and results should be interpreted as broad comparative patterns rather than hypothesis-driven estimates.

In conclusion, our study, based on a granular approach, offers valuable insights into the evolving patterns of suicide mortality across Europe. While substantial declines in suicide rates over the past decades reflect important social and material progress, concerning reversals in specific populations across the lifespan underscore the need for multidisciplinary and tailored prevention and intervention strategies. The observed variations in suicide trajectories across age, sex and country reflect how suicide risk evolves in response to shifting social, economic, and cultural dynamics. This risk manifests differently across age groups, ranging from mental health challenges linked to emotional and social pressures in youth, to combined psychosocial and economic stressors in midlife, and to cognitive and physical decline and social isolation in later life. These patterns highlight the need for age-specific and life-course approaches for adequate and equitable suicidal prevention, including strengthening youth and community-based mental health services, reducing barriers to care in working-age adults, and improving primary care screening and support, especially for older populations.

## Supporting information

10.1192/j.eurpsy.2025.10137.sm001 Bertuccio et al. supplementary material Bertuccio et al. supplementary material

## Data Availability

This study used publicly available datasets, which provide aggregated cause-of-death data by country, sex, age group, and calendar year (https://www.who.int/data/data-collection-tools/who-mortality-database). When population denominators were missing in the WHO, data were retrieved from the United Nations World Population Prospects (https://www.un.org/development/desa/pd/data-landing-page), which provides demographic estimates. Specific datasets obtained through the elaborations in this study are available upon reasonable request by research-oriented institutions. All authors had full access to all the data in the study and accept responsibility to submit for publication.

## Supplementary material

The supplementary material for this article can be found at http://doi.org/10.1192/j.eurpsy.2025.10137.
