# Age- and sex-patterns of suicide trends in Europe: 1990–2022 comparative analysis of official WHO mortality data – CORRIGENDUM

**DOI:** 10.1192/j.eurpsy.2026.10149

**Published:** 2026-01-20

**Authors:** Paola Bertuccio, Andrea Amerio, Giansanto Mosconi, Enrico Grande, Carlo La Vecchia, Alessandra Costanza, Giacomo Pietro Vigezzi, Riccardo Vecchio, Andrea Aguglia, Isabella Berardelli, Gianluca Serafini, Mario Amore, Maurizio Pompili, Anna Odone

**Keywords:** adults, elderly, mortality, suicide, young

This article was originally published with an error in the author list. The name of the first author was given as Paola Bertuccico. This should have read Paola Bertuccio. The authors apologise for this error, and it has now been corrected.
