# Supplementary material for: Age- and sex-patterns of suicide trends in Europe: 1990–2022 comparative analysis of official WHO mortality data
Source: Eur Psychiatry. 2025 Dec 2;68(1):e173. doi: 10.1192/j.eurpsy.2025.10137 (PMC12721994; doi:10.1192/j.eurpsy.2025.10137)
Supplement: Bertuccio et al. supplementary material [file S0924933825101375sup001.pdf]

Age- and sex-patterns of suicide trends in Europe: 1990-2022 comparative analysis of official WHO mortality data

Contents

Figure S1. Age-specific suicide mortality rates in 2020-2022 (or according to data availability) by country, separately by sex........ 2

Figure S2. Age-specific suicide mortality rates in 2020 by geographical areas, separately by sex........ 3

Table S1. Age-specific suicide mortality rates per 100,000 among males, in four periods over the 1990-2022 period, and corresponding percentage differences: 1990 (1990-1994) vs 2000 (2000-2004), 2000 (2000-2004) vs 2010 (2010-2014), and 2010 (2010-2014) vs 2020 (2020-2022), separately by country........ 4

Table S2. Age-specific suicide mortality rates per 100,000 among females, in four periods over the 1990-2022 period, and corresponding percentage differences: 1990 (1990-1994) vs 2000 (2000-2004), 2000 (2000-2004) vs 2010 (2010-2014), and 2010 (2010-2014) vs 2020 (2020-2022), separately by country and geographical area........ 8

Figure S1. Age-specific suicide mortality rates in 2020-2022 (or according to data availability) by country, separately by sex.

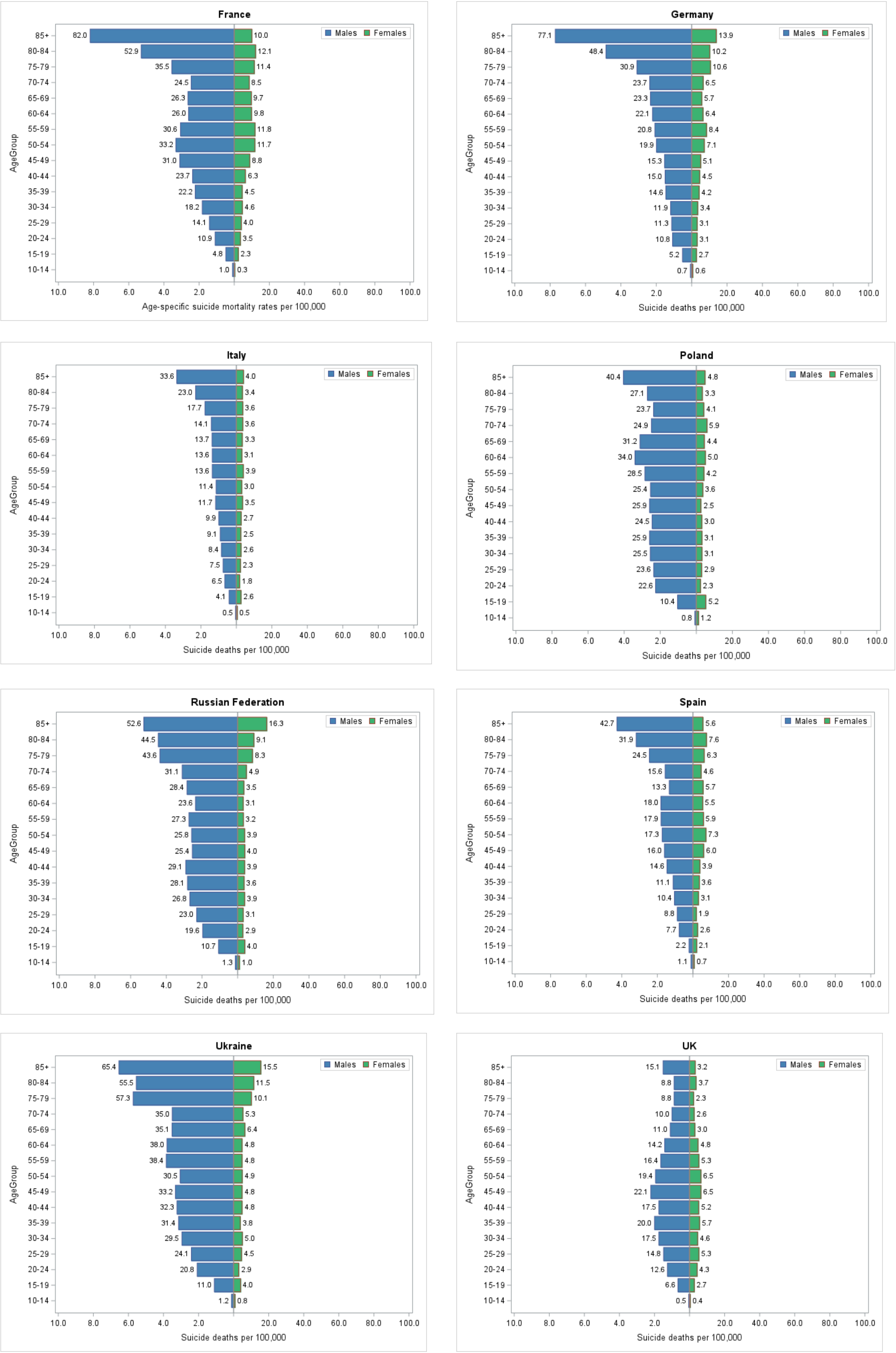

Figure S2. Age-specific suicide mortality rates in 2020 by geographical areas, separately by sex.

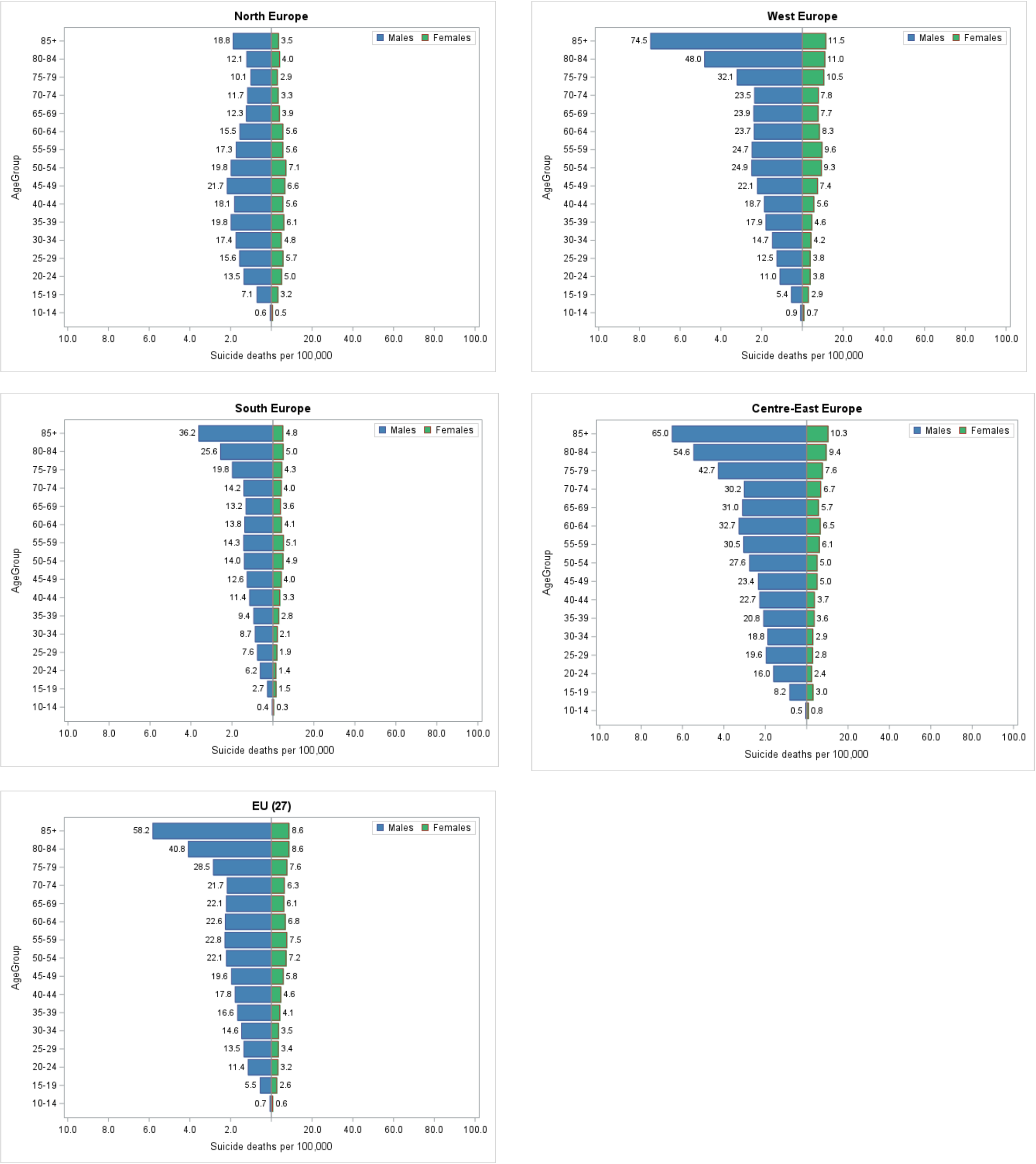

Table S1. Age-specific suicide mortality rates per 100,000 among males, in four periods over the 1990-2022 period, and corresponding percentage differences: 1990 (1990-1994) vs 2000 (2000-2004), 2000 (2000-2004) vs 2010 (2010-2014), and 2010 (2010-2014) vs 2020 (2020-2022), separately by country. Increases are coloured in red, while decreases in green.

		Age-specific suicide mortality rate/100,000				Δ%		
	Age Group	1990–1994	2000–2004	2010–2014	2020–2022[1,2]	1990–2000	2000–2010	2010–2020
France	*10-14*	0.84	1.20	0.97	0.94	43.8	-19.3	-3.2
	15-19	7.76	6.99	5.42	4.46	-10.0	-22.5	-17.7
	20-24	22.58	15.72	11.55	11.27	-30.4	-26.6	-2.4
	25-29	28.80	20.60	16.21	14.06	-28.5	-21.3	-13.2
	30-34	35.83	27.56	20.41	17.99	-23.1	-25.9	-11.9
	35-39	40.51	33.94	26.12	22.45	-16.2	-23.1	-14.0
	40-44	41.22	39.14	32.44	24.42	-5.0	-17.1	-24.7
	45-49	39.59	40.04	36.86	29.41	1.1	-8.0	-20.2
	50-54	41.09	33.83	36.77	31.55	-17.7	8.7	-14.2
	55-59	38.39	29.14	32.06	30.78	-24.1	10.0	-4.0
	60-64	37.50	29.44	27.29	28.61	-21.5	-7.3	4.9
	65-69	44.85	33.43	25.90	27.09	-25.5	-22.5	4.6
	70-74	51.77	40.58	31.99	27.75	-21.6	-21.2	-13.3
	75-79	72.50	53.91	44.92	35.88	-25.6	-16.7	-20.1
	80-84	107.90	70.50	59.21	51.14	-34.7	-16.0	-13.6
	85+	147.22	116.32	88.52	81.50	-21.0	-23.9	-7.9
Germany	*10-14*	1.28	0.90	0.71	0.73	-29.8	-21.2	3.6
	15-19	9.00	8.45	6.21	5.21	-6.1	-26.6	-16.1
	20-24	17.07	14.73	11.88	10.81	-13.7	-19.4	-9.0
	25-29	20.02	15.12	13.73	11.29	-24.5	-9.2	-17.8
	30-34	23.11	16.65	14.26	11.88	-28.0	-14.3	-16.7
	35-39	25.21	19.72	14.88	14.56	-21.7	-24.6	-2.2
	40-44	26.75	23.84	17.92	14.99	-10.9	-24.8	-16.3
	45-49	30.14	25.77	20.91	15.27	-14.5	-18.8	-27.0
	50-54	32.99	26.32	23.52	19.94	-20.2	-10.6	-15.2
	55-59	33.09	25.88	24.47	20.84	-21.8	-5.5	-14.8
	60-64	32.69	25.57	23.06	22.10	-21.8	-9.8	-4.1
	65-69	33.11	26.64	23.76	23.33	-19.5	-10.8	-1.8
	70-74	41.69	32.70	28.81	23.73	-21.6	-11.9	-17.6
	75-79	65.60	44.17	34.72	30.88	-32.7	-21.4	-11.1
	80-84	93.27	61.29	49.25	48.38	-34.3	-19.6	-1.8
	85+	121.97	96.21	75.78	77.11	-21.1	-21.2	1.8
Italy	*10-14*	0.84	0.49	0.40	0.41	-42.6	-17.5	2.6
	15-19	3.83	3.69	2.96	3.61	-3.7	-19.9	22.2
	20-24	8.93	8.38	7.04	6.31	-6.1	-16.0	-10.4
	25-29	10.61	9.78	8.56	7.22	-7.8	-12.5	-15.7
	30-34	11.25	10.39	8.92	8.20	-7.6	-14.2	-8.0
	35-39	10.87	9.88	10.04	9.02	-9.1	1.6	-10.2
	40-44	11.16	11.56	11.01	9.89	3.6	-4.7	-10.1
	45-49	11.79	11.85	13.11	11.36	0.5	10.7	-13.3
	50-54	13.89	12.27	14.32	11.71	-11.7	16.7	-18.2
	55-59	15.59	12.32	13.94	13.49	-21.0	13.2	-3.2
	60-64	18.71	14.00	14.01	12.80	-25.2	0.1	-8.6
	65-69	21.08	15.00	13.80	13.01	-28.9	-8.0	-5.7
	70-74	25.70	20.65	16.07	13.26	-19.6	-22.2	-17.5
	75-79	36.29	25.42	20.62	19.60	-30.0	-18.9	-5.0
	80-84	48.03	35.59	25.33	22.92	-25.9	-28.8	-9.5
	85+	60.28	47.78	33.57	33.09	-20.7	-29.8	-1.4
Poland	*10-14*	2.17	2.02	1.32	0.62	-6.8	-34.8	-53.1
	15-19	12.13	14.36	14.27	10.75	18.4	-0.6	-24.7
	20-24	21.74	25.33	25.70	21.58	16.5	1.5	-16.0
	25-29	26.83	24.93	24.77	24.33	-7.0	-0.7	-1.8
	30-34	32.17	28.29	26.30	25.33	-12.1	-7.0	-3.7

	Age Group	Age-specific suicide mortality rate/100,000 1990–1994	2000–2004	2010–2014	2020–2022[1,2]	Δ% 1990–2000	2000–2010	2010–2020
	35-39	36.81	34.57	30.49	25.13	-6.1	-11.8	-17.6
	40-44	41.05	41.86	38.87	25.48	2.0	-7.2	-34.4
	45-49	42.84	46.27	43.56	25.76	8.0	-5.9	-40.8
	50-54	45.25	46.53	51.29	28.64	2.8	10.2	-44.2
	55-59	39.52	41.54	52.37	32.76	5.1	26.1	-37.4
	60-64	33.94	36.73	42.49	32.34	8.2	15.7	-23.9
	65-69	33.37	33.00	37.11	26.14	-1.1	12.5	-29.6
	70-74	31.77	31.40	33.75	22.98	-1.1	7.5	-31.9
	75-79	31.29	28.94	30.13	25.85	-7.5	4.1	-14.2
	80-84	31.14	30.74	30.97	27.18	-1.3	0.7	-12.2
	85+	32.77	38.79	39.27	35.54	18.4	1.2	-9.5
Russian Federation	*10-14*	5.29	6.19	3.03	1.65	17.0	-51.0	-45.4
	15-19	28.11	36.36	21.31	12.20	29.4	-41.4	-42.7
	20-24	45.89	73.86	41.11	24.64	60.9	-44.3	-40.1
	25-29	62.95	84.65	48.23	30.00	34.5	-43.0	-37.8
	30-34	76.91	81.07	51.62	34.13	5.4	-36.3	-33.9
	35-39	82.37	85.31	49.13	36.08	3.6	-42.4	-26.6
	40-44	86.26	91.24	44.84	33.98	5.8	-50.8	-24.2
	45-49	93.65	98.21	45.97	31.08	4.9	-53.2	-32.4
	50-54	98.05	97.66	45.14	31.94	-0.4	-53.8	-29.3
	55-59	90.61	89.38	42.78	31.44	-1.4	-52.1	-26.5
	60-64	80.55	81.11	34.97	27.95	0.7	-56.9	-20.1
	65-69	74.95	85.63	39.35	29.99	14.3	-54.0	-23.8
	70-74	76.98	97.45	52.43	34.12	26.6	-46.2	-34.9
	75-79	92.19	82.62	63.21	47.86	-10.4	-23.5	-24.3
	80-84	105.94	82.78	76.73	51.03	-21.9	-7.3	-33.5
	85+	120.43	103.10	74.56	66.23	-14.4	-27.7	-11.2
Spain	*10-14*	0.93	0.61	0.32	0.74	-35.0	-47.8	134.5
	15-19	4.47	3.62	2.89	2.69	-19.1	-20.0	-7.1
	20-24	9.87	8.95	6.38	7.27	-9.3	-28.7	13.9
	25-29	11.66	10.67	6.73	8.13	-8.5	-36.9	20.8
	30-34	11.17	12.05	8.89	10.65	7.8	-26.2	19.8
	35-39	10.46	12.75	10.30	10.97	21.9	-19.2	6.5
	40-44	9.97	12.18	13.73	14.30	22.2	12.7	4.1
	45-49	12.08	13.02	14.85	15.80	7.8	14.1	6.4
	50-54	13.96	13.78	16.96	17.76	-1.3	23.1	4.7
	55-59	17.20	13.59	15.15	18.03	-21.0	11.5	19.0
	60-64	17.95	14.89	14.57	17.97	-17.0	-2.2	23.4
	65-69	20.75	18.73	14.50	14.89	-9.7	-22.6	2.7
	70-74	26.34	23.07	18.63	17.37	-12.4	-19.2	-6.7
	75-79	38.63	33.12	26.41	21.36	-14.3	-20.3	-19.1
	80-84	49.84	42.70	34.02	31.84	-14.3	-20.3	-6.4
	85+	60.74	59.75	41.41	44.30	-1.6	-30.7	7.0
UK	*10-14*	0.32	0.20	0.32	0.48	-36.8	59.5	50.9
	15-19	6.11	5.06	4.82	6.60	-17.3	-4.7	37.0
	20-24	16.91	12.75	10.79	12.55	-24.6	-15.4	16.4
	25-29	17.59	15.58	11.96	14.81	-11.5	-23.2	23.7
	30-34	17.01	16.91	14.21	17.47	-0.6	-16.0	23.0
	35-39	17.76	17.05	16.30	20.02	-4.0	-4.4	22.8
	40-44	18.53	16.40	19.14	17.49	-11.5	16.7	-8.6
	45-49	17.70	15.89	19.10	22.14	-10.2	20.2	15.9
	50-54	15.85	14.11	18.07	19.39	-11.0	28.0	7.3
	55-59	14.75	11.91	15.76	16.35	-19.2	32.4	3.7
	60-64	11.96	9.74	12.23	14.22	-18.6	25.6	16.3
	65-69	12.14	8.89	8.72	10.96	-26.8	-1.8	25.6
	70-74	11.96	9.38	9.21	10.04	-21.5	-1.9	9.1
	75-79	14.27	10.23	9.47	8.80	-28.3	-7.4	-7.1
	80-84	18.59	12.70	11.24	8.83	-31.7	-11.5	-21.5

	Age Group	Age-specific suicide mortality rate/100,000 1990–1994	2000–2004	2010–2014	2020–2022[1,2]	Δ% 1990–2000	2000–2010	2010–2020
	85+	22.22	18.08	13.62	15.09	-18.7	-24.7	10.8
Ukraine	*10-14*	3.19	3.65	3.24	2.00	14.5	-11.1	-38.4
	15-19	14.24	17.05	16.32	12.23	19.7	-4.3	-25.1
	20-24	25.02	37.64	33.32	23.04	50.5	-11.5	-30.8
	25-29	37.12	45.49	37.47	26.73	22.6	-17.6	-28.7
	30-34	47.64	51.29	41.06	30.31	7.7	-19.9	-26.2
	35-39	52.99	58.22	40.77	32.41	9.9	-30.0	-20.5
	40-44	58.45	66.02	42.00	32.88	13.0	-36.4	-21.7
	45-49	67.17	74.85	44.75	34.86	11.4	-40.2	-22.1
	50-54	72.85	77.95	45.73	35.18	7.0	-41.3	-23.1
	55-59	62.91	76.26	45.15	38.00	21.2	-40.8	-15.8
	60-64	62.40	68.86	38.61	36.95	10.4	-43.9	-4.3
	65-69	57.78	67.18	44.31	39.14	16.3	-34.1	-11.7
	70-74	55.60	77.38	54.95	45.93	39.2	-29.0	-16.4
	75-79	71.92	67.51	55.87	59.63	-6.1	-17.2	6.7
	80-84	86.81	70.40	75.13	58.39	-18.9	6.7	-22.3
	85+	107.93	90.65	74.29	66.28	-16.0	-18.1	-10.8
EU (27)	*10-14*	1.47	1.27	0.83	0.68	-13.5	-34.7	-17.6
	15-19	8.43	8.41	6.97	5.49	-0.2	-17.2	-21.2
	20-24	17.20	16.00	13.67	11.35	-6.9	-14.6	-17.0
	25-29	21.04	17.38	15.04	13.47	-17.4	-13.4	-10.4
	30-34	25.07	19.92	16.20	14.62	-20.5	-18.7	-9.7
	35-39	28.58	23.31	18.59	16.62	-18.5	-20.2	-10.6
	40-44	30.57	28.57	22.53	17.84	-6.5	-21.1	-20.8
	45-49	31.92	31.34	25.90	19.57	-1.8	-17.4	-24.4
	50-54	33.23	30.29	29.04	22.06	-8.8	-4.1	-24.0
	55-59	32.51	27.41	29.47	22.76	-15.7	7.5	-22.8
	60-64	31.65	26.37	25.41	22.62	-16.7	-3.6	-11.0
	65-69	34.30	27.34	23.45	22.15	-20.3	-14.2	-5.6
	70-74	39.92	32.38	27.08	21.66	-18.9	-16.4	-20.0
	75-79	54.85	40.18	32.87	28.48	-26.7	-18.2	-13.3
	80-84	75.32	53.00	42.40	40.76	-29.6	-20.0	-3.9
	85+	99.79	79.52	58.99	58.15	-20.3	-25.8	-1.4
North Europe	*10-14*	0.59	0.36	0.42	0.60	-38.2	16.4	42.0
	15-19	7.70	6.62	5.92	7.11	-14.0	-10.5	19.9
	20-24	18.81	15.44	12.62	13.52	-17.9	-18.3	7.1
	25-29	20.11	17.04	13.62	15.62	-15.3	-20.1	14.7
	30-34	19.69	18.03	15.35	17.41	-8.4	-14.8	13.4
	35-39	21.35	18.65	16.77	19.82	-12.7	-10.1	18.2
	40-44	22.69	18.86	19.80	18.10	-16.9	5.0	-8.6
	45-49	21.94	18.71	20.42	21.68	-14.7	9.1	6.2
	50-54	20.42	17.16	19.98	19.75	-16.0	16.4	-1.1
	55-59	18.91	14.83	18.09	17.29	-21.6	22.0	-4.4
	60-64	16.08	13.01	14.56	15.53	-19.1	11.9	6.6
	65-69	16.18	11.95	11.30	12.26	-26.1	-5.4	8.5
	70-74	16.50	12.88	11.65	11.70	-21.9	-9.6	0.4
	75-79	20.74	13.97	12.59	10.13	-32.6	-9.9	-19.5
	80-84	24.90	17.11	14.33	12.11	-31.3	-16.2	-15.5
	85+	30.26	24.61	17.60	18.81	-18.7	-28.5	6.9
West Europe	*10-14*	1.08	1.02	0.88	0.89	-5.3	-14.2	1.5
	15-19	8.86	8.37	6.42	5.37	-5.6	-23.2	-16.4
	20-24	20.17	16.02	12.35	11.00	-20.6	-22.9	-10.9
	25-29	23.46	18.35	15.03	12.52	-21.8	-18.1	-16.7
	30-34	27.59	21.16	16.78	14.68	-23.3	-20.7	-12.5
	35-39	30.80	25.00	19.87	17.94	-18.8	-20.5	-9.7
	40-44	32.27	29.37	24.22	18.68	-9.0	-17.5	-22.9
	45-49	33.13	31.20	27.06	22.08	-5.8	-13.3	-18.4
	50-54	34.48	29.52	28.62	24.91	-14.4	-3.1	-12.9

	Age Group	Age-specific suicide mortality rate/100,000				Δ%		
		1990–1994	2000–2004	2010–2014	2020–2022[1,2]	1990–2000	2000–2010	2010–2020
	55-59	34.38	27.42	27.86	24.72	-20.3	1.6	-11.3
	60-64	34.13	26.73	25.05	23.70	-21.7	-6.3	-5.4
	65-69	37.72	28.99	24.07	23.85	-23.1	-17.0	-0.9
	70-74	45.36	35.62	29.25	23.54	-21.5	-17.9	-19.5
	75-79	66.58	47.38	37.55	32.15	-28.8	-20.8	-14.4
	80-84	96.70	64.11	51.99	47.95	-33.7	-18.9	-7.8
	85+	130.08	103.93	78.34	74.52	-20.1	-24.6	-4.9
South Europe	*10-14*	0.86	0.49	0.33	0.36	-42.6	-33.1	8.6
	15-19	3.88	3.44	2.83	2.71	-11.4	-17.7	-4.3
	20-24	8.95	8.04	6.42	6.17	-10.2	-20.1	-3.9
	25-29	10.83	9.67	7.48	7.62	-10.7	-22.7	2.0
	30-34	10.93	10.60	8.71	8.72	-3.0	-17.9	0.1
	35-39	10.32	10.72	10.00	9.37	3.9	-6.7	-6.3
	40-44	10.31	11.50	12.23	11.43	11.6	6.4	-6.5
	45-49	11.47	11.92	13.80	12.61	4.0	15.8	-8.7
	50-54	13.37	12.38	15.48	13.97	-7.4	25.0	-9.7
	55-59	15.79	12.45	14.70	14.33	-21.1	18.0	-2.5
	60-64	17.44	14.11	14.48	13.84	-19.1	2.6	-4.4
	65-69	20.29	15.96	14.16	13.18	-21.3	-11.3	-7.0
	70-74	25.22	21.07	17.26	14.22	-16.5	-18.1	-17.6
	75-79	35.42	27.67	23.21	19.84	-21.9	-16.1	-14.5
	80-84	47.03	37.16	29.51	25.62	-21.0	-20.6	-13.2
	85+	56.97	51.46	37.17	36.21	-9.7	-27.8	-2.6
Centre-East Europe	*10-14*	2.73	2.71	1.77	0.46	-0.9	-34.7	-74.3
	15-19	12.72	13.57	12.55	8.17	6.7	-7.5	-34.9
	20-24	22.70	27.04	23.84	15.98	19.1	-11.8	-33.0
	25-29	31.38	31.03	25.60	19.63	-1.1	-17.5	-23.3
	30-34	39.26	35.90	27.70	18.77	-8.6	-22.8	-32.2
	35-39	44.19	43.39	30.67	20.84	-1.8	-29.3	-32.1
	40-44	49.47	52.31	35.51	22.68	5.8	-32.1	-36.1
	45-49	56.23	57.57	41.67	23.37	2.4	-27.6	-43.9
	50-54	61.13	58.17	46.41	27.61	-4.8	-20.2	-40.5
	55-59	53.87	55.06	47.36	30.48	2.2	-14.0	-35.6
	60-64	50.01	51.60	38.83	32.72	3.2	-24.7	-15.7
	65-69	49.91	49.52	38.23	30.95	-0.8	-22.8	-19.0
	70-74	51.77	55.98	44.04	30.17	8.1	-21.3	-31.5
	75-79	64.80	56.30	45.97	42.66	-13.1	-18.4	-7.2
	80-84	77.48	66.96	57.76	54.62	-13.6	-13.7	-5.4
	85+	99.55	93.57	66.97	64.96	-6.0	-28.4	-3.0

[1]Last year: 2022 for France, Poland, and Spain; 2021 for Italy; 2020 for Germany, the UK, and all geographical areas; 2019 for the Russian Federation and Ukraine.
[2]Age-specific suicide mortality rates in the 2015-2019 period were shown for the Russian Federation and Ukraine.

Table S2. Age-specific suicide mortality rates per 100,000 among females, in four periods over the 1990-2022 period, and corresponding percentage differences: 1990 (1990-1994) vs 2000 (2000-2004), 2000 (2000-2004) vs 2010 (2010-2014), and 2010 (2010-2014) vs 2020 (2020-2022), separately by country and geographical area.Increases are coloured in red, while decreases in green.

		Age-specific suicide mortality rate/100,000				Δ%		
	Age Group	1990–1994	2000–2004	2010–2014	2020–2022[1,2]	1990–2000	2000–2010	2010–2020
France	*10-14*	0.24	0.42	0.64	0.75	72.9	52.3	17.7
	15-19	2.90	2.75	2.30	2.95	-5.1	-16.4	28.3
	20-24	5.87	3.91	3.29	4.13	-33.4	-15.8	25.2
	25-29	7.83	5.02	3.88	4.15	-35.9	-22.7	7.0
	30-34	10.21	7.43	4.43	4.66	-27.3	-40.4	5.1
	35-39	11.73	9.61	6.49	4.66	-18.1	-32.4	-28.2
	40-44	14.16	12.81	9.46	6.41	-9.6	-26.2	-32.2
	45-49	15.21	14.03	11.51	8.20	-7.7	-18.0	-28.7
	50-54	17.73	14.21	12.65	11.47	-19.9	-11.0	-9.3
	55-59	18.11	13.42	11.93	10.87	-25.9	-11.1	-8.8
	60-64	16.72	12.87	10.59	9.70	-23.0	-17.7	-8.5
	65-69	17.13	12.17	9.42	9.34	-28.9	-22.6	-0.9
	70-74	17.93	12.73	10.74	8.80	-29.0	-15.7	-18.0
	75-79	24.85	13.20	10.87	9.26	-46.9	-17.7	-14.7
	80-84	23.58	14.97	11.34	12.10	-36.5	-24.3	6.7
	85+	25.65	16.41	12.05	10.89	-36.0	-26.6	-9.7
Germany	*10-14*	0.44	0.35	0.47	0.55	-20.8	34.6	18.4
	15-19	2.67	2.51	2.33	2.66	-6.0	-7.3	14.1
	20-24	4.39	3.30	3.07	3.11	-24.7	-7.0	1.4
	25-29	5.37	3.92	3.31	3.08	-27.0	-15.6	-6.9
	30-34	6.50	3.99	4.07	3.43	-38.6	2.1	-15.9
	35-39	7.21	5.46	4.03	4.19	-24.3	-26.3	3.9
	40-44	8.32	6.50	5.60	4.53	-21.9	-13.9	-19.0
	45-49	11.34	7.62	7.09	5.06	-32.9	-7.0	-28.5
	50-54	13.01	8.33	8.15	7.15	-36.0	-2.1	-12.4
	55-59	12.57	9.39	7.79	8.39	-25.3	-17.1	7.7
	60-64	13.84	9.55	7.21	6.39	-31.0	-24.5	-11.4
	65-69	15.61	9.64	8.79	5.67	-38.3	-8.8	-35.5
	70-74	19.11	11.15	9.06	6.54	-41.6	-18.7	-27.9
	75-79	25.01	13.48	10.13	10.59	-46.1	-24.8	4.5
	80-84	28.61	18.30	11.92	10.25	-36.0	-34.9	-14.0
	85+	28.06	22.33	15.12	13.91	-20.4	-32.3	-8.0
Italy	*10-14*	0.37	0.25	0.25	0.25	-32.6	0.1	2.3
	15-19	1.26	1.27	1.18	2.06	0.3	-7.0	75.0
	20-24	2.12	1.60	1.61	1.46	-24.6	0.9	-9.5
	25-29	2.63	2.20	1.68	1.75	-16.6	-23.4	4.2
	30-34	3.33	2.62	2.07	2.29	-21.2	-21.0	10.4
	35-39	3.44	3.02	2.63	2.36	-12.2	-13.0	-10.1
	40-44	3.89	3.46	3.08	2.61	-11.1	-10.9	-15.2
	45-49	4.33	3.59	3.57	3.46	-17.1	-0.6	-3.1
	50-54	5.19	4.31	4.14	3.54	-17.1	-3.8	-14.6
	55-59	5.97	4.48	3.93	3.68	-24.9	-12.3	-6.4
	60-64	6.91	4.52	3.71	3.15	-34.6	-18.0	-15.1
	65-69	7.18	5.19	3.83	3.29	-27.7	-26.1	-14.3
	70-74	8.77	5.29	4.40	3.46	-39.6	-16.9	-21.4
	75-79	8.96	6.29	4.32	3.42	-29.9	-31.3	-20.8
	80-84	9.31	6.33	4.28	3.91	-32.0	-32.5	-8.7
	85+	9.21	5.92	3.64	4.06	-35.7	-38.5	11.6
Poland	*10-14*	0.35	0.53	0.54	1.61	53.9	1.5	197.3
	15-19	2.74	2.52	2.79	4.59	-7.9	10.8	64.5
	20-24	2.65	2.77	2.21	2.69	4.5	-20.3	21.7
	25-29	3.37	2.72	2.05	3.10	-19.3	-24.6	50.9
	30-34	5.36	3.47	2.68	3.38	-35.2	-22.8	26.2
	35-39	6.39	5.13	3.29	3.25	-19.8	-35.9	-1.3

	Age Group	Age-specific suicide mortality rate/100,000 1990–1994	2000–2004	2010–2014	2020–2022[1,2]	Δ% 1990–2000	2000–2010	2010–2020
	40-44	7.15	6.52	4.38	3.17	-8.9	-32.8	-27.5
	45-49	7.70	8.73	6.33	3.80	13.4	-27.5	-39.9
	50-54	8.24	8.27	7.45	4.25	0.5	-10.0	-42.9
	55-59	7.87	7.25	7.58	4.40	-7.8	4.5	-41.9
	60-64	8.11	7.67	7.52	4.61	-5.4	-2.0	-38.7
	65-69	8.13	6.72	6.18	4.78	-17.4	-8.0	-22.6
	70-74	7.91	6.66	5.01	3.64	-15.8	-24.8	-27.2
	75-79	7.43	6.40	5.29	3.27	-13.9	-17.3	-38.2
	80-84	6.42	6.47	5.02	4.41	0.8	-22.5	-12.1
	85+	5.71	6.05	5.21	3.67	6.0	-13.8	-29.6
Russian Federation	*10-14*	1.28	1.75	1.77	1.27	36.8	0.9	-28.0
	15-19	7.40	8.04	6.82	4.70	8.7	-15.2	-31.1
	20-24	7.66	9.63	6.66	3.73	25.7	-30.8	-44.0
	25-29	8.45	10.41	6.82	4.10	23.2	-34.5	-39.9
	30-34	9.36	10.78	7.21	4.77	15.2	-33.1	-33.9
	35-39	11.18	10.98	7.08	4.93	-1.8	-35.5	-30.4
	40-44	13.20	12.23	7.00	4.95	-7.3	-42.7	-29.3
	45-49	15.83	12.81	7.19	5.20	-19.1	-43.9	-27.6
	50-54	17.56	13.16	6.64	4.80	-25.0	-49.5	-27.7
	55-59	17.05	12.11	5.89	4.06	-29.0	-51.4	-31.1
	60-64	19.25	13.95	6.24	4.22	-27.5	-55.3	-32.3
	65-69	20.71	15.30	7.54	4.67	-26.1	-50.7	-38.0
	70-74	24.19	19.73	9.71	6.08	-18.4	-50.8	-37.3
	75-79	28.91	22.81	13.43	9.47	-21.1	-41.1	-29.5
	80-84	33.12	30.23	17.75	11.07	-8.7	-41.3	-37.6
	85+	37.00	38.17	25.24	17.27	3.2	-33.9	-31.6
Spain	*10-14*	0.26	0.18	0.21	0.49	-32.0	17.2	140.0
	15-19	1.25	1.35	1.28	2.05	8.6	-5.0	59.5
	20-24	2.20	1.96	1.57	2.51	-11.3	-19.6	59.9
	25-29	2.66	2.28	1.69	2.86	-14.2	-25.8	69.1
	30-34	2.70	3.16	2.44	2.79	17.0	-22.9	14.4
	35-39	3.35	3.19	3.38	3.80	-4.7	6.1	12.4
	40-44	2.75	3.99	4.45	4.08	45.2	11.5	-8.3
	45-49	3.33	4.41	4.50	5.52	32.5	2.0	22.8
	50-54	4.61	4.71	5.16	6.98	2.2	9.5	35.2
	55-59	5.93	4.54	4.58	6.89	-23.5	0.8	50.4
	60-64	5.87	5.62	4.47	5.58	-4.2	-20.5	24.9
	65-69	7.96	6.33	5.36	5.45	-20.5	-15.3	1.6
	70-74	9.68	6.67	5.94	5.91	-31.1	-10.9	-0.5
	75-79	10.74	7.18	5.48	5.78	-33.2	-23.8	5.5
	80-84	11.10	8.34	5.36	7.64	-24.9	-35.7	42.4
	85+	11.71	9.31	5.62	5.97	-20.5	-39.6	6.1
UK	*10-14*	0.08	0.16	0.22	0.41	95.9	38.8	84.8
	15-19	1.49	1.66	1.64	2.74	11.0	-1.0	67.0
	20-24	2.82	3.05	2.74	4.29	8.1	-10.1	56.4
	25-29	3.77	3.48	3.09	5.32	-7.8	-11.1	72.1
	30-34	4.13	3.70	3.35	4.61	-10.4	-9.4	37.5
	35-39	3.84	4.16	4.20	5.71	8.4	1.0	35.9
	40-44	4.63	4.24	4.34	5.20	-8.4	2.5	19.8
	45-49	5.31	4.75	4.95	6.46	-10.4	4.1	30.5
	50-54	4.77	4.60	5.22	6.51	-3.5	13.6	24.7
	55-59	4.67	4.26	4.23	5.28	-8.8	-0.7	24.9
	60-64	5.18	3.53	3.40	4.79	-31.8	-3.7	40.9
	65-69	5.28	3.33	2.77	3.01	-37.0	-16.7	8.5
	70-74	5.22	3.45	2.76	2.62	-33.9	-20.1	-4.8
	75-79	5.91	3.78	2.70	2.33	-36.1	-28.6	-13.7
	80-84	5.90	3.28	2.93	3.72	-44.4	-10.7	27.2
	85+	5.35	4.38	2.98	3.17	-18.0	-32.1	6.5

	Age Group	Age-specific suicide mortality rate/100,000 1990–1994	2000–2004	2010–2014	2020–2022[1,2]	Δ% 1990–2000	2000–2010	2010–2020
Ukraine	*10-14*	0.80	0.96	1.76	1.16	20.8	83.0	-34.3
	15-19	4.63	3.53	5.22	4.13	-23.7	47.7	-20.9
	20-24	4.65	4.44	4.66	3.75	-4.4	4.9	-19.6
	25-29	5.58	5.65	4.66	4.34	1.1	-17.5	-6.7
	30-34	5.78	7.29	5.45	4.93	26.2	-25.3	-9.4
	35-39	7.65	7.70	6.36	4.51	0.5	-17.4	-29.0
	40-44	9.26	8.74	6.96	5.30	-5.7	-20.3	-23.8
	45-49	11.63	9.52	6.88	5.53	-18.1	-27.8	-19.6
	50-54	13.88	10.77	7.01	5.96	-22.4	-35.0	-14.9
	55-59	12.93	11.95	6.68	5.53	-7.6	-44.1	-17.2
	60-64	15.48	12.51	7.80	5.48	-19.2	-37.6	-29.8
	65-69	16.10	12.99	10.29	6.69	-19.3	-20.7	-35.0
	70-74	17.34	15.60	11.12	7.81	-10.0	-28.8	-29.8
	75-79	22.21	16.54	12.28	9.71	-25.6	-25.7	-20.9
	80-84	25.51	21.74	17.88	12.08	-14.8	-17.8	-32.5
	85+	28.24	27.51	18.41	15.15	-2.6	-33.1	-17.7
EU (27)	*10-14*	0.39	0.47	0.45	0.58	19.3	-2.6	27.6
	15-19	2.54	2.51	2.37	2.65	-1.1	-5.7	11.6
	20-24	4.12	3.05	2.86	3.23	-25.9	-6.4	13.1
	25-29	5.06	3.54	3.09	3.39	-30.0	-12.7	9.6
	30-34	6.49	4.47	3.53	3.53	-31.1	-21.1	0.0
	35-39	7.65	5.88	4.28	4.05	-23.1	-27.1	-5.4
	40-44	8.66	7.31	5.72	4.60	-15.6	-21.8	-19.5
	45-49	10.25	8.72	7.23	5.78	-14.9	-17.0	-20.1
	50-54	11.42	9.14	8.22	7.23	-19.9	-10.1	-12.0
	55-59	11.58	8.83	7.96	7.53	-23.8	-9.9	-5.4
	60-64	12.09	9.10	7.28	6.82	-24.7	-20.0	-6.4
	65-69	13.34	8.89	7.33	6.10	-33.3	-17.6	-16.7
	70-74	15.48	9.97	8.00	6.34	-35.6	-19.7	-20.7
	75-79	18.57	11.20	8.13	7.64	-39.7	-27.4	-6.1
	80-84	20.84	13.37	8.79	8.56	-35.8	-34.2	-2.6
	85+	21.03	14.92	9.91	8.61	-29.1	-33.6	-13.2
North Europe	*10-14*	0.15	0.26	0.30	0.46	75.1	14.1	56.7
	15-19	1.96	2.19	2.20	3.16	11.9	0.3	43.6
	20-24	3.41	3.64	3.47	5.00	6.8	-4.7	43.9
	25-29	4.43	4.02	3.65	5.66	-9.3	-9.4	55.2
	30-34	5.13	4.13	3.98	4.78	-19.5	-3.7	20.0
	35-39	5.22	4.86	4.61	6.11	-7.0	-5.0	32.6
	40-44	6.38	5.22	4.89	5.60	-18.2	-6.4	14.6
	45-49	7.48	5.97	5.78	6.58	-20.1	-3.2	13.9
	50-54	7.07	5.93	6.39	7.06	-16.1	7.7	10.6
	55-59	6.86	5.74	5.27	5.61	-16.3	-8.2	6.5
	60-64	7.05	4.96	4.61	5.60	-29.6	-7.1	21.5
	65-69	7.27	4.25	3.87	3.85	-41.5	-9.1	-0.3
	70-74	7.16	4.59	3.64	3.29	-35.9	-20.6	-9.6
	75-79	8.05	4.82	3.38	2.90	-40.1	-30.0	-14.2
	80-84	7.42	4.34	3.71	4.01	-41.5	-14.5	7.9
	85+	6.22	5.30	3.62	3.49	-14.7	-31.7	-3.5
West Europe	*10-14*	0.39	0.44	0.52	0.68	14.6	16.9	30.3
	15-19	2.81	2.87	2.58	2.85	2.1	-10.3	10.6
	20-24	5.27	3.79	3.40	3.81	-28.1	-10.3	12.0
	25-29	6.47	4.72	4.02	3.80	-27.0	-15.0	-5.4
	30-34	8.38	5.64	4.50	4.24	-32.7	-20.2	-5.8
	35-39	9.62	7.54	5.50	4.60	-21.7	-27.0	-16.4
	40-44	11.27	9.35	7.47	5.59	-17.0	-20.1	-25.2
	45-49	13.04	10.80	9.33	7.43	-17.2	-13.6	-20.4
	50-54	14.57	11.38	10.54	9.34	-21.9	-7.4	-11.4
	55-59	14.72	11.55	10.23	9.60	-21.5	-11.4	-6.2

	Age Group	Age-specific suicide mortality rate/100,000				Δ%		
		1990–1994	2000–2004	2010–2014	2020–2022[1,2]	1990–2000	2000–2010	2010–2020
	60-64	14.96	11.16	9.06	8.26	-25.4	-18.8	-8.8
	65-69	15.94	10.82	9.27	7.71	-32.1	-14.3	-16.8
	70-74	18.31	12.22	9.88	7.79	-33.3	-19.1	-21.2
	75-79	23.61	13.52	10.34	10.49	-42.8	-23.5	1.5
	80-84	25.66	16.75	11.35	10.99	-34.7	-32.3	-3.1
	85+	25.45	19.67	13.06	11.49	-22.7	-33.6	-12.0
South Europe	*10-14*	0.28	0.22	0.21	0.28	-22.1	-2.1	29.0
	15-19	1.26	1.25	1.17	1.45	-1.2	-6.8	24.5
	20-24	2.06	1.60	1.57	1.41	-22.4	-2.0	-10.5
	25-29	2.60	2.11	1.63	1.87	-18.9	-22.8	14.9
	30-34	2.98	2.75	2.27	2.15	-7.7	-17.2	-5.6
	35-39	3.22	2.92	2.84	2.84	-9.3	-2.9	-0.1
	40-44	3.29	3.42	3.51	3.28	3.8	2.6	-6.4
	45-49	3.85	3.71	3.84	3.97	-3.8	3.6	3.4
	50-54	4.76	4.20	4.34	4.88	-11.7	3.2	12.6
	55-59	5.56	4.21	4.20	5.13	-24.2	-0.1	22.0
	60-64	6.15	4.77	3.84	4.11	-22.5	-19.5	7.2
	65-69	7.19	5.34	4.40	3.60	-25.7	-17.5	-18.3
	70-74	8.40	5.44	4.91	4.01	-35.2	-9.8	-18.3
	75-79	9.19	6.47	4.80	4.34	-29.6	-25.8	-9.5
	80-84	9.64	7.07	4.85	5.01	-26.7	-31.4	3.3
	85+	9.93	6.96	4.55	4.77	-29.9	-34.7	4.9
Centre-East Europe	*10-14*	0.65	0.75	0.87	0.83	15.5	15.0	-4.4
	15-19	3.84	3.05	3.30	3.00	-20.6	8.2	-9.0
	20-24	4.45	3.50	3.03	2.42	-21.4	-13.3	-20.2
	25-29	5.17	4.07	3.33	2.77	-21.3	-18.1	-16.8
	30-34	6.24	5.37	3.79	2.88	-14.0	-29.4	-24.0
	35-39	8.01	6.70	4.65	3.56	-16.4	-30.6	-23.4
	40-44	9.20	8.17	5.90	3.69	-11.2	-27.7	-37.4
	45-49	11.52	9.89	7.32	5.01	-14.1	-26.0	-31.6
	50-54	13.21	10.42	7.90	5.01	-21.1	-24.3	-36.5
	55-59	12.75	10.42	7.90	6.08	-18.3	-24.2	-23.0
	60-64	14.44	11.68	7.95	6.46	-19.2	-31.9	-18.7
	65-69	15.56	11.81	8.36	5.67	-24.1	-29.2	-32.2
	70-74	18.00	13.99	9.77	6.71	-22.3	-30.2	-31.2
	75-79	21.61	15.52	10.40	7.62	-28.2	-33.0	-26.7
	80-84	24.80	19.45	12.80	9.45	-21.6	-34.2	-26.2
	85+	26.98	23.71	14.40	10.28	-12.1	-39.3	-28.6

[1]Last year: 2022 for France, Poland, and Spain; 2021 for Italy; 2020 for Germany, the UK, and all geographical areas; 2019 for the Russian Federation and Ukraine.
[2]Age-specific suicide mortality rates in the 2015-2019 period were shown for the Russian Federation and Ukraine.
